# Non-Newtonian Nano-Fluids in Blasius and Sakiadis Flows Influenced by Magnetic Field

**DOI:** 10.3390/nano12234254

**Published:** 2022-11-29

**Authors:** Imran Abbas, Shahid Hasnain, Nawal A. Alatawi, Muhammad Saqib, Daoud S. Mashat

**Affiliations:** 1 Department of Mathematics, Faculty of Science, Air University, Islamabad Campus 44000, Pakistan; 2 Department of Mathematics, Faculty of Science, King Abdulaziz University, Jeddah 21589, Saudi Arabia; 3 Department of Mathematics, Khwaja Fareed University of Engineering & Information Technology, Rahim Yaar Khan 48800, Pakistan

**Keywords:** Newtonian & non-Newtonian nano-fluids, permeable surface, suction & injection, Sakiadis & Blasius flows, similarity transformation, Runge-Kutta Fehlberg (RKF45)

## Abstract

Current study solves heat transfer and fluid flow problem in Newtonian and non-Newtonian nano-fluids through a permeable surface with a magnetic field effects which is done in the presence of injection and suction for the first time. In order to solve the governing partial differential equations numerically, we used the Runge-Kutta Fehlberg (RKF45) technique in which the similarity transformation method is applied. This approach converts the governing partial differential equations into ordinary differential equations. In this particular investigation nano-particles of copper, copper oxide, titanium dioxide, and aluminium oxide are studied by considering CMC/water as a base fluid with the effect of magnetic field on the classical Blasius and Sakiadis flows of nano-fluids. Validation is carried out using the previously obtained numerical findings. We looked at the power-law index (*n*), the volume fraction (φ) of nano-particles and the permeability parameter ($f_{w}$) which affects the flow of nano-fluid and the transfer of heat. Non-Newtonian nano-fluid demonstrates superior performance in terms of heat transfer when compared to Newtonian nano-fluid in both the injection and the impermeable surfaces. Altering the nano-particles’ composition, on the other hand, has a far greater impact on the heat transfer process that occurs during suction. Graphics show the impacts of governing physical parameters on Blasius and Sakiadis flow velocity, temperature, skin friction coefficient, and reduced Nusselt number. Physical and engineering interest are explored in detail.

## 1. Introduction

Non-Newtonian fluids have been the focus of study on drag force and energy transfer over the last half century. This is because, the widespread usage trend of applications using Non-Newtonian and Newtonian fluids within the manufacturing sector, such as molten polymers, slurries, pulps and emulsions. The increasing demands of contemporary technologies, in the realms of petrochemicals, electricity generation, and microelectronics need to create more efficient fluids of different sorts for according to the efficiency of the heat transfer. Nano-fluids are made by dispersing solid particles on the nano-scale scale into low viscosity base liquids such as water, ethylene glycol (EG), and oils all have high heat conductivity. Therefore, it has become difficult to find appropriate nano-fluids with enhanced heat transfer capabilities and high thermal conductivity. Thermal conductivity of oxide nano-fluids has been the subject of several published investigations such as, Cu, $TiO_{2}$, $SiO_{2}$ and $Al_{2}O_{3}$. In these investigations, a research on the relationship between solid content and thermal conductivity was carried out. Nano-particles enhance fluids thermal conductivity significantly, which in turn improves the heat transfer properties. Therefore, dispersing micro/nano sized particle materials in liquid boosts the thermal conductivity of the fluids. Viscosity increment is due to addition of nano-particles which reduces the thermal advantages of nano-fluids by necessitating more pumping power in the systems. Nano-fluids’ thermal behaviour is one of the most essential issues, however the most important problem is the stability of the nano-fluids which is still difficult to achieve the appropriate level of stability in today’s world. Although reducing the concentration of nano-fluids is the best method to preserve excellent fluidity, most studies have instead concentrated on the suspension stability of nano-fluids with no significant outcomes. Non-Newtonian fluids give platform to describe the rheological characteristics of nano-fluids by the constitutive equations which are based on empirical or semi-empirical formulae. In addition, the non-linear terms of the associated differential systems are increased by the presence of additional rheological factors in such interactions. To this end, the literature presents a wide variety of non-Newtonian fluid models (each with its own unique rheological effects) [1,2,3,4,5,6].

Sui et al. [7] studied the use of power law model for non-Newtonian fluids with boundary layer (BL) using mixed convection mechanism to get insights about heat transfer. A moving conveyor along an inclined plate was proposed by them, using the homotopy analysis (HAM)technique, they conducted in depth the numerical simulations of the temperature profile and the effects of power-law viscosity and shear flow. Numerical study of the heat transfer using fluids with shear thickening behaviour on permeable stretching plane which give rise to BL flow, done by Jalil et al. [8]. They founded accurate analytical and numerical solutions to the similarity equations in non-linear case of study. The numerical investigation done by Ahmed et al. [9] along with boundary layer theory, by using power law fluid which placed on stretched sheet for fluid flow and transfer of heat. In order to get analytical and numerical solutions to the proposed equations, they made use of the homotopy (HAM) technique coupled with shooting method. Guha et al. [10] took into consideration free convection boundary layer flow as part of their investigation. The finite difference time marching method was the approach which used to solve the problem. Meghdad et al. [11] considered fluid with power law nature over flat vertical surface in case of two dimensional problem for fluid flow and transfer of heat. They proposed, a non-linear stretching of the surface which was coupled with consideration of heat radiation, to investigate the influence of buoyancy parameter on mixed convection. Fluid which pass over a porous and flat plate, considered by Kishan et al. [12] to solve BL problem with help of finite difference (FD) schemes while same group carried out the numerical analysis to power law fluid by considering convection with mixed nature. A power law fluid along the impact of magnetic field, effect of radiation, heat generation/absorption and thermal dispersion on permeable flat and stretched plates, with viscous dissipation effects are studied to investigate heat transfer and fluid flow problem [13,14].

Ram Reddy et al. [15] considered porous medium or non-darcy material to performed a computational simulation for a sloping, inclined plate. A study of a power law non-Newtonian fluid flowing over a moving surface on surface slip and heat absorption or generation in heat transfer and fluid flow. Such researched heat transport in non-Newtonian, non-uniform fluids. A slip boundary layer and viscous dissipation at a specific permeable surface employing power law fluid is analysed. Temperature drop and velocity slip in a pseudo-plastic fluid moving across a porous surface in a magnetic field are observed [16,17,18,19]. Although many articles have been written about the fluid flow and the heat transfer due to non-Newtonian fluids over variant surfaces (permeable) which devoid of nano-particles because their authors were unaware of the existence of the newer field of study. Choi invented the term nano-fluid to describe a novel class of heat transfer fluids made by floating metallic nano-particles in ordinary fluids. When base fluids are compared with other nano-fluids for heat transfer indicate the improvement in thermal conductivity and convective heat transfer [20,21,22,23,24,25,26,27,28]. Water, ethylene glycol, oil, and other heat transfer fluids are used as base fluids. Nano-particles are made from metallic (like copper), metal oxide, carbon-based, or combinations of these elements [29,30,31,32,33,34,35]. Non-Newtonian nano-fluids with assumption of Brownian motion during the simulation of flow, mass, and heat transfer which included a transverse magnetic field, injection, and suction over a permeable stretched sheet, investigated by Sandeep et al. [36]. Non-Newtonian nano-fluids flow over stretching sheet with non-linear properties which was subject of research of Madhu et al. [37]. The non-Newtonian behaviours and heat transfer brought about by the introduction of a magnetic field are the primary topic that are covered in this article. Therefore, a literature concentrated on certain fields and applications is viewable from inside the literature [38,39,40,41,42,43].

Nano-fluids flow over horizontal plate with Sakiadis and Blasius effects, are the focus of all of the investigations that have been discussed so far. However, as of yet, no contribution has been made on non-Newtonian (NN) nano-fluids (NFs) considering magnetic field over surface which is a porous, because of the nano-particles that consist of Cu, CuO, $TiO_{2}$, and $Al_{2}O_{3}$. Therefore, the mixture of sodium carboxymethyl cellulose (CMC) and water used as a base fluid for current research. In Sakiadis and Blasius flows, energy, continuity and momentum equations are governing system which is the main focus of this work. The use of an appropriate similarity transformation and then solved numerically through MATLAB. In the next section, we explore further into the formation patterns with physical and rheological features of nano-fluids along the mathematical formulation and method of solution and discussion.

### 1.1. Rheological and Thermal Characteristics of Nano-Fluids

Solids dispersed in liquids enhance heat conductivity, since from 1900s, scientists and engineers have added solid particles to liquids to increase their low heat conductivity. The increment in thermal conductivity of solid-dispersed liquids in which size of particles was restricted to microns or millimetres and solid particle concentration was predicted by Maxwell and Yang [44,45,46]. Over the past decades, several organisations and corporations have studied nano-fluids for their potential to improve heat transfer coefficients. The traditional Maxwell theory or the H-C [35] model did not predict the thermal conductivity anomaly. Many hypothesised processes explain nano-fluids’ heat transfer phenomena however, these models were inconsistent and limiting in scope, until Maxwell proposition which was proposed in 19th century about suspension thermal conductivity [46]. Such model reveals that the solid-liquid combination has better thermal conductivity than the base fluid which make a viable heat transfer in fluids. Thermal conductivity is greater in solids than liquids, therefore, the mixture of solids and liquids may settle, clog, foul, erode and cause excessive pressure decrease. Hence, nanotechnology solves these challenges which increase interests in researchers to study nano-particles and heat transfer in fluids. Researchers employ many formulae to determine the thermal conductivity of nano-fluids. Mehrali et al. [47], discussed a comparative study of Nusselt numbers, although the measured thermal conductivities exhibited relatively large deviations from the published correlations. Therefore, changes in thermal conductivity show less severe effects on the modulation of the transport behaviour of nano-fluids [48,49,50]. For current research work, CMC/water is used as the base fluid (recommendation) having Pr=6.2, to study fluid flow and heat transfer. According to Oztop et al. [51], nano-fluids characteristics are as follows:

(1) $$\begin{array}{c} \alpha_{nf}=\frac{k_{nf}}{{\left(\rho C_{p}\right)}_{nf}},\mu_{nf}=\frac{u}{{\left(1-\varphi \right)}^{2.5}},\frac{k_{nf}}{k_{f}}=\frac{\left(k_{s}+2k_{f}\right)-2\varphi \left(k_{f}-k_{s}\right)}{\left(k_{s}+2k_{f}\right)+\varphi \left(k_{f}+k_{s}\right)}, \end{array}$$

(2) $$\begin{array}{c} {\left(\rho C_{p}\right)}_{nf}=\left(1-\varphi \right){\left(\rho C_{p}\right)}_{f}+\varphi \left(\rho C_{p}\right),\rho_{nf}=\left(1-\varphi \right)\rho_{f}+\varphi \rho_{s}. \end{array}$$

Water, ethylene glycol, and transformer oil have lower thermal conductivity than nano-structures. Researchers previously reported that the thermal conductivity of nano-fluids was influenced by several parameters. Base fluids type, particle size, particle shape, fluids and temperature are some examples of such parameters [3,49,50,52,53]. Table 1 summarizes the results of thermal conductivity measurement conducted over past 15 years by different research centres.

#### Effectiveness of Heat Transfer in Nano-Fluids

For nano-fluids used in heat exchange equipment, heat transfer coefficient is superior than thermal conductivity using different nano-particles and base fluids. Therefore, nano-fluids heat transfer has two theories in which the first school of thoughts says that the heat transfer coefficient may be increased without compromising pumping power. The second school of thinking says heat transfer coefficient increases are restricted and mitigated by increased pumping power. MIT and Helmut Schmidt University reflect the second school of thinking. Both groups’ findings are in Table 2.

Shriram et al. [55] explored $Al_{2}O_{3}$-water nano-fluids as heat exchanger coolants. Experiments were done at various Reynolds numbers and nano-particle volume concentrations. Therefore, adding nano-particles to the base fluid (CuO-water) increased heat transfer coefficients such that, Khairul et al. [56] tested nano-fluids in a corrugated plate heat exchanger. Water-CuO nano-fluids improved the heat transfer coefficient from 18.50% to 27.20% when nano-particle volume concentration was 0.50% to 1.50%. A Peclet number of about 40,000, Yang et al. [57] evaluated the heat transfer in a circular pipe. Local convective heat transfer improved. Nano-particles and fluid temperature enhanced heat transfer rates. Brownian motion and reduced thermal boundary layer improve heat transfer. Researchers are studying the heat transfer performance of nano-fluids in pipe flow, including natural and induced convection, after Choi et al. [3] reported that nano-sized solid particles boost the thermal conductivity of base fluids.

## 2. Mathematical Formulation

Consider the two dimensional and steady state boundary layer (BL) flow over a plate which is flat and permeable. Therefore, the plate is immersed in sodium carboxymethyl cellulose (CMC) nano-fluid, considered as base fluid. The temperature and velocity of the free stream are denoted by $T_{\infty }$ and $U_{\infty }$. The CMC and water combination, is a non-Newtonian pseudo-plastic fluid. Table 3 has a listing of the viscosity of CMC/water, along with its other features. Experiments show that the thermo-physical properties of CMC/water with a concentration of less than 6% and water with a concentration of 0% are quite similar to one another. Table 4 has a listing of the thermo-physical parameters of the nano-fluids that were taken into consideration. The model takes into consideration a flow regime that is laminar and incompressible. In addition, one of the presumptions is that there is no slippage between the fluid phase and the nano-particles which are in a condition of thermal equilibrium. The continuity, momentum and energy equations constitutes governing system. A magnetic field of strength $B_{0}$ that is consistent and applied in a normal direction to the plate. Because it is presumed to be negligible in comparison to the magnetic field that is being applied, the induced magnetic field is ignored.

In the governing system, the flow velocity is presented by $u^{*}$ in *x* and $v^{*}$ in *y* directions respectively. The power-law number is indicated by the letter *n* in the exponent, which is connected to the base fluid. Both Newtonian fluids and pseudo-plastic fluids have *n* values that are either 1 or between 0 and 1, depending on the kind of fluids. Additionally, the value of *n* for dilatant fluids is greater than 1. The mass transfer velocity, denoted here by $V_{w}$, is something that ought to be thought of as being next to the plate. In the case of an impermeable plate, $V_{w}$ equals 0. When there is blowing or injection present, $V_{w}$ has a positive value, but when there is suction present, it has a negative value. The plate becomes heated from the bottom up by a process known as convection, in which a fluid whose temperature is equal to $T_{f}$ and whose heat transfer coefficient is $h_{f}$ is responsible for the heating. The governing equations of motion and heat transfer for the classical Blasius and Sakiadis flow of nano-fluids could well be represented using the Boussinesq and boundary layer approximations which are as follows:

Governing Blasius flow:

(3) $$\begin{array}{c} \frac{\partial u^{*}}{\partial x}+\frac{\partial v^{*}}{\partial y}=0, \end{array}$$

(4) $$\begin{array}{c} u^{*}\frac{\partial u^{*}}{\partial x}+v^{*}\frac{\partial u^{*}}{\partial y}=\frac{\mu_{nf}}{\rho_{nf}}\frac{\partial }{\partial y}\left(|\frac{\partial u^{*}}{\partial y}{|}^{n-1}\frac{\partial u^{*}}{\partial y}\right)-\frac{\sigma_{nf}B_{0}^{2}}{\rho_{nf}}\left(u^{*}-U_{\infty }\right), \end{array}$$

(5) $$\begin{array}{c} u^{*}\frac{\partial T^{*}}{\partial x}+v^{*}\frac{\partial T^{*}}{\partial y}=\alpha_{nf}\frac{\partial }{\partial y}\left(|\frac{\partial T^{*}}{\partial y}{|}^{n-1}\frac{\partial T^{*}}{\partial y}\right)+\frac{Q_{0}}{\rho c_{p}}\left(T^{*}-T_{\infty }\right). \end{array}$$

Boundary conditions for Blasius flow:

(6) $$\begin{array}{c} u^{*}\to U_{\infty }\;\;\;as\;\;\;y\to \infty ,\;u^{*}=0,\;\;v^{*}=V_{W}\left(x\right)\;\;\;as\;\;\;y=0. \end{array}$$

(7) $$\begin{array}{c} -k_{nf}\frac{\partial T^{*}}{\partial y}=h_{f}\left(T_{f}-T_{w}\right)\;\;\;at\;\;\;y=0,\;T^{*}\to T_{\infty }\;\;\;as\;\;\;y\to \infty . \end{array}$$

Boundary conditions for Sakiadis flow:

(8) $$\begin{array}{c} u^{*}\to U_{\infty }\;\;\;as\;\;\;y\to \infty ,\;u^{*}=U_{w},\;\;v^{*}=V_{W}\left(x\right)\;\;\;at\;\;\;y=0. \end{array}$$

(9) $$\begin{array}{c} -k_{nf}\frac{\partial T^{*}}{\partial y}=h_{f}\left(T_{f}-T_{w}\right)\;\;\;at\;\;\;y=0,\;T^{*}\to T_{\infty }\;\;\;as\;\;\;y\to \infty . \end{array}$$

Nano-fluids density is represented by $\rho_{nf}$ while the thermal conductivity is scale down under $\alpha_{nf}$.

## 3. Solution Methodology

The following similarity transformations have been developed in order to facilitate a more straightforward mathematical analysis of our research:

(10) $$\begin{array}{c} \psi ={\left(U_{\infty }^{2n-1}vf_{x}\right)}^{\frac{1}{n+1}}f\left(\eta \right),\;u^{*}=\frac{\partial \psi }{\partial y},\;\;\;v^{*}=-\frac{\partial \psi }{\partial x}, \end{array}$$

(11) $$\begin{array}{c} \eta ={\left(\frac{U_{\infty }^{2-n}}{v_{f}x}\right)}^{\frac{1}{n+1}}y,\;\;\;\theta =\frac{T^{*}-T_{\infty }}{T_{f}-T_{\infty }} \end{array}$$

Therefore, Blasius and Sakiadis’ momentum and energy equations with constitutive boundary conditions are;

Governing Blasius flow:

(12) $$\begin{array}{c} \frac{1}{A_{4}A_{1}}\left(\right|f^{\prime \prime }{{|}^{n-1}f^{\prime \prime })}^{\prime }+\frac{1}{n+1}f^{\prime \prime }f-M^{2}\frac{\sigma_{nf}}{\sigma_{f}}\left(f^{\prime }-1\right)=0 \end{array}$$

(13) $$\begin{array}{c} -\frac{A_{3}}{Pr_{f}A_{2}}\left(\right|f^{\prime \prime }{{|}^{n-1}\theta^{\prime })}^{\prime }+\frac{1}{n+1}f\theta^{\prime }\pm \lambda \theta =0 \end{array}$$

where

(14) $$\begin{array}{c} \frac{\sigma_{nf}}{\sigma_{f}}=1+\frac{3\varphi \left(\frac{\sigma_{s}}{\sigma_{f}}\right)-1}{\left(\frac{\sigma_{s}}{\sigma_{f}}+2\right)-\varphi \left(\frac{\sigma_{s}}{\sigma_{f}}\right)-1},\\ M=\sqrt{\left(\frac{xB_{0}^{2}}{\rho_{nf}}\right)}\;\;and\;\;\lambda =\frac{Q_{0}}{\rho C_{p}} \end{array}$$

Boundary conditions:

(15) $$\begin{array}{c} f\left(0\right)=f_{w},\;\;\;f^{\prime }\left(0\right)=0,\;\;\;f^{\prime }\left(\infty \right)=1, \end{array}$$

(16) $$\begin{array}{c} \theta^{\prime }\left(0\right)=A_{3}a\left(1-\theta \left(0\right)\right),\;\;\;\;\theta \left(\infty \right)=0. \end{array}$$

Governing Sakiadis flow:

(17) $$\begin{array}{c} \frac{1}{A_{4}A_{1}}\left(\right|f^{\prime \prime }{{|}^{n-1}f^{\prime \prime })}^{\prime }+\frac{1}{n+1}f^{\prime \prime }f-M^{2}\frac{\sigma_{nf}}{\sigma_{f}}f^{\prime }=0 \end{array}$$

(18) $$\begin{array}{c} -\frac{A_{3}}{Pr_{f}A_{2}}\left(\right|f^{\prime \prime }{{|}^{n-1}\theta^{\prime })}^{\prime }+\frac{1}{n+1}f\theta^{\prime }\pm \lambda \theta =0 \end{array}$$

Boundary conditions:

(19) $$\begin{array}{c} f\left(0\right)=-f_{w},\;\;\;f^{\prime }\left(0\right)=0,\;\;\;f^{\prime }\left(\infty \right)=0, \end{array}$$

(20) $$\begin{array}{c} \theta^{\prime }\left(0\right)=A_{3}a\left(1-\theta \left(0\right)\right),\;\;\;\theta \left(\infty \right)=0. \end{array}$$

where $f_{w}=0,1,-1$ stands for impermeable sheet suction and injection respectively.

(21) $$\begin{array}{c} A_{1}=\frac{1}{\left(1-\varphi \right)+\frac{\varphi \rho_{s}}{\rho_{f}}},\;\;\;A_{2}=-\frac{1}{\left(1-\varphi \right)+\varphi \frac{{\left(\rho C_{p}\right)}_{s}}{{\left(\rho C_{p}\right)}_{f}}}, \end{array}$$

(22) $$\begin{array}{c} A_{3}=-\frac{\left(\frac{k_{s}}{k_{f}}+2\right)-2\varphi \left(1-\frac{k_{s}}{k_{f}}\right)}{\left(\frac{k_{s}}{k_{f}}+2\right)+\varphi \left(1-\frac{k_{s}}{k_{f}}\right)},\;\;\;A_{4}=\frac{1}{{\left(1-\varphi \right)}^{2.5}}. \end{array}$$

## 4. Important Engineering Parameters

In this investigation, the local skin friction coefficient $C_{f}$ and the local Nusselt number $Nu_{x}$ are considered to be of significant physical consequence, and they can be defined as follows:

(23) $$\begin{array}{c} C_{fx}=-\frac{2\tau_{w}}{\rho_{f}U^{2}},\;\;\;\;Nu_{x}=\frac{xq_{w}}{k_{f}\left(T_{w}-T_{\infty }\right)}, \end{array}$$

where

(24) $$\begin{array}{c} \tau_{w}=\mu_{nf}{\left(|\frac{\partial u^{*}}{\partial y}{|}^{n-1}\frac{\partial u^{*}}{\partial y}\right)}_{y=0},\;\;\;q_{w}=-k_{nf}{\left(\frac{\partial T^{*}}{\partial y}\right)}_{y=0}. \end{array}$$

By employing similarity transformation, the obtained results are as follows:

(25) $$\begin{array}{c} Cf_{x}Re_{x}^{\frac{1}{n+1}}=-2\frac{\mu_{nf}}{\mu_{f}}f^{\prime \prime }\left(0\right){\left|f^{\prime \prime }\left(0\right)\right|}^{n-1},\\ Nu_{x}Re_{x}^{\frac{-1}{n+1}}=-\frac{k_{nf}}{k_{f}}\theta^{\prime }\left(0\right). \end{array}$$

## 5. Discussion

In this part, the effects of several factors, such as different kind of nano-material, permeability, volume fraction (VF) related to solid particles, index for the power law, magnetic strength, and the source term parameter, has been discussed. For each nano-fluid, the findings are presented with regard to the non-dimensionlized temperature and the velocity fields. The following contains an analysis as well as a discussion of the aforementioned findings.

### 5.1. Verification

**Figure 1:**
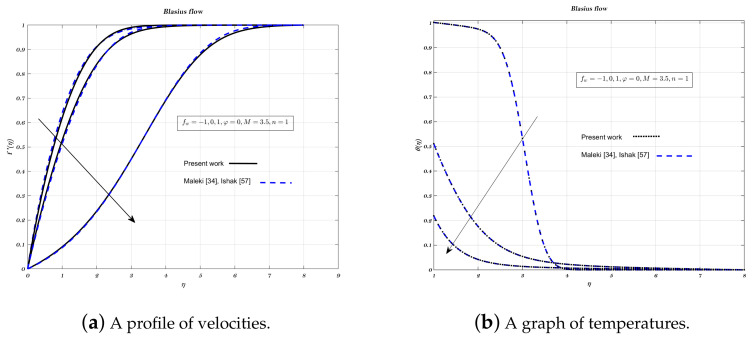
Impact of different permeability on non-dimensionalized profiles for velocity and temperature.

**Figure 2:**
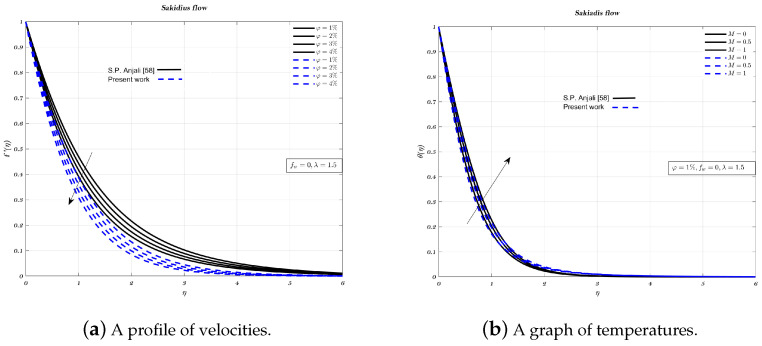
Impact of different volume fraction on non-dimensionalized velocity and parameter *M* on temperature profiles.

Results from the present experiment are compared and analysed against those from the studies conducted by Maleki and Ishak [32,60], for each combination of φ and $f_{w}$. In order to test the correctness of the current numerical work, non-dimensional temperature and velocity profiles give confirmation in case of Blasius flow which can be seen from Figure 1 and Figure 2a,b for Sakiadis flow. It is clear from analysis, that there is a high level of concordance between the old findings [32,60]. It denotes that the existing numerical approach has an excellent accuracy level.

### 5.2. The Impact of Investigated Factors on the Flow Velocity Profile

**Figure 3:**
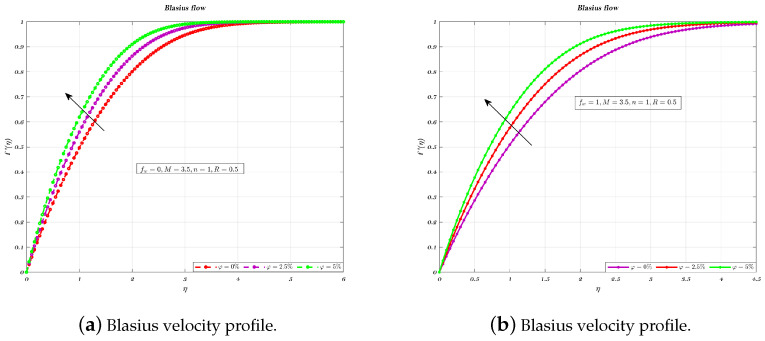
Different VF on non-dimensionalized velocity, by considering $f_{w}=0$ & $f_{w}=1$.

**Figure 4:**
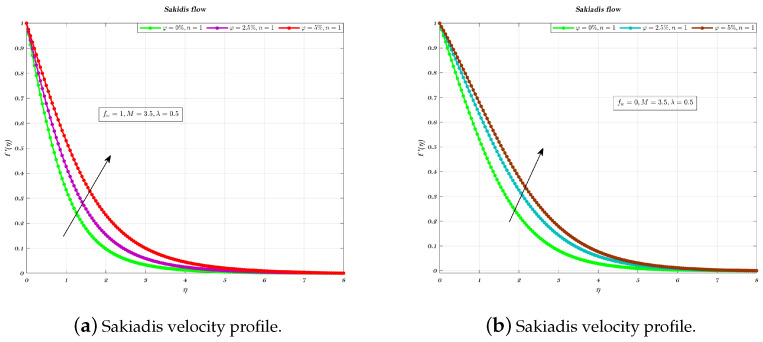
Different VF on non-dimensionalized velocity, by considering $f_{w}=1$ & $f_{w}=0$.

Figure 3a,b and Figure 4a,b illustrate the influence of nano-particles volume fraction for two different values of $f_{w}$ such as for 0 and 1, which correspond to an impermeable surface and suction, respectively. It has been shown that the fluid velocity increased as the VF which is related to nano particles increased, therefore holds true for both suction and impermeable surfaces [11,31,32,60]. In addition, the employment of Newtonian nano-fluids for suction and an impermeable plate causes a drop in thickness of the boundary layer (BL) but has no effect on the velocity gradient across the plate [32,60].

**Figure 5:**
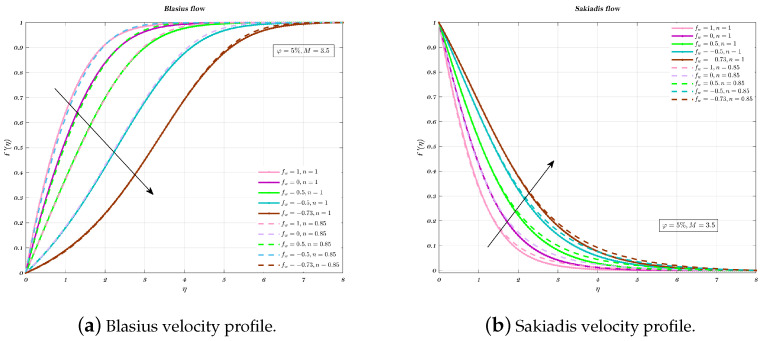
Impact of different permeability, by considering both Newtonian and non-Newtonian cases.

Figure 5a,b illustrate, for both Newtonian and non-Newtonian nano-fluids, the impact of permeability on velocity profile. Because the boundary layer development is regulated by suction, the velocity decreases in proportion to the suction parameter. This is consistent with the fact that suction does stabilise the growth of the boundary layer. Injection, on the other hand, produces greater fluid diffusion and extends the boundary layer. This is shown by the fact that the boundary layer area expands as a result of the injection.

**Figure 6:**
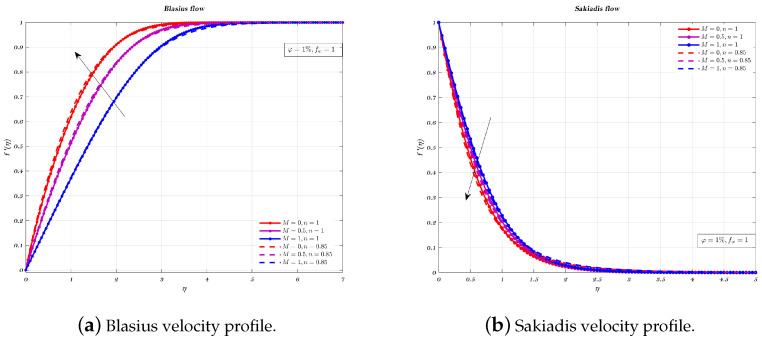
Impact of different values of parameter *M* on both Newtonian and non-Newtonian cases for Blasius and Sakiadis flows.

The Blasius flow of Newtonian and non-Newtonian nano-fluids for different values of the magnetic interaction parameter *M* to analyse non-dimensional velocity, can be seen from Figure 6a. It has been shown without a reasonable doubt that, for both kinds of nano-fluids, the velocity of the nano-fluid rises with an increase in the intensity of the magnetic field. The thickness of the boundary layer will decrease in response to an increase in the intensity of the magnetic field. Therefore, the nano-fluid whose motion is slowed down. Because of this, the velocity of the nano-fluid will rise for both kinds of nano-fluids when the parameter *M* is increased, the flow will be described as being in a Blasius configuration.

Figure 6b illustrates, the magnetic interaction parameter *M* affects the dimensionless velocity when the Sakiadis flow is considered for both the nano-fluids under consideration. It has been shown that an increase in the magnetic field has the effect of slowing down the velocity. This is due to the fact that the magnetic field in transverse direction generates a resistive force, known as Lorentz force which cut down the hydrodynamic BL thickness for both nano-fluids.

### 5.3. The Impact of Investigated Factors on the Temperature Profile

**Figure 7:**
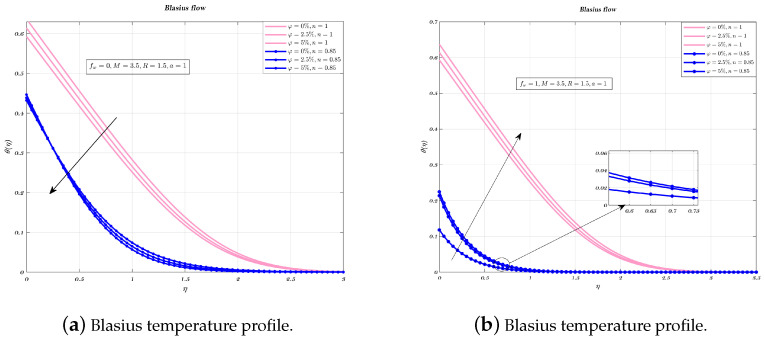
Impact of different volume fraction on non-dimensionalized temperature, by considering $f_{w}=0,\;1$.

**Figure 8:**
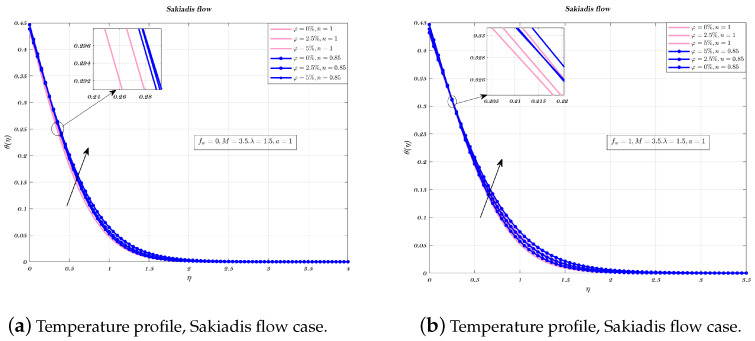
Impact of different volume fraction on non-dimensionalized temperature, by considering $f_{w}=0,\;1$ for both Newtonian and non-Newtonian cases.

Temperature profiles are shown for an impermeable surface, as well as for suction, as seen in the Figure 7 and Figure 8a,b. The decremental status of the dimensionless temperature is because of nano-particles VF in the suction and in the impermeable plate case, however, this decrease is followed by an increase. When an impermeable plate is combined with a non-Newtonian fluid and the addition of nano-particles to increase their volume fraction, results in decrease in temperature. When the plate is subjected to suction, VF does not have significant effects on the surface of the plate. In every instance, the thickness of the BL was reduced when a non-Newtonian fluid is used.

**Figure 9:**
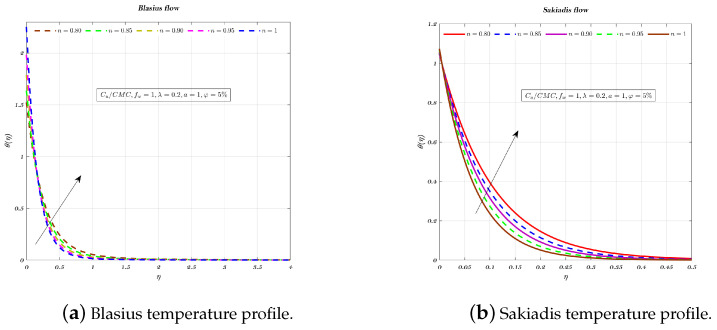
Impact of different values of *n* on non-dimensionalized temperature, by considering $f_{w}=1$ for both flows.

**Figure 10:**
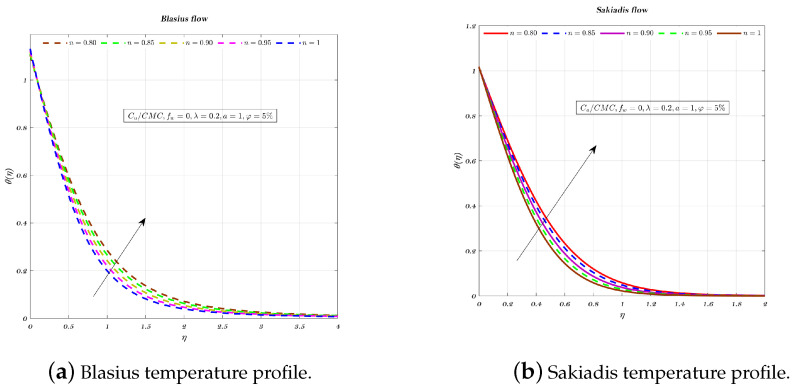
Impact of different values of *n* on non-dimensionalized temperature, by considering $f_{w}=0$ for both flows.

**Figure 11:**
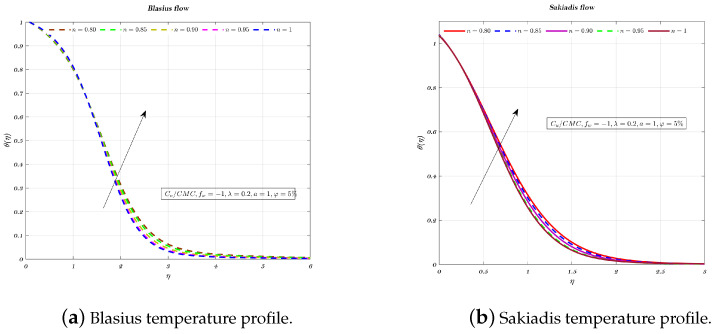
Impact of different values of *n* on non-dimensionalized temperature, by considering $f_{w}=-1$ for both flows.

Figure 9, Figure 10 and Figure 11a,b depict the temperature profile for various values of the index *n* which is related to the power law for an impermeable surface, suction, and injection. It can be shown that the non-dimensional temperature rises with increasing *n* for suction and impermeable surface, but this tendency is not apparent for injection. Injection temperatures do not follow this pattern. Additionally, the thickness of the BL grows in every circumstance as the power-law index rises.

**Figure 12:**
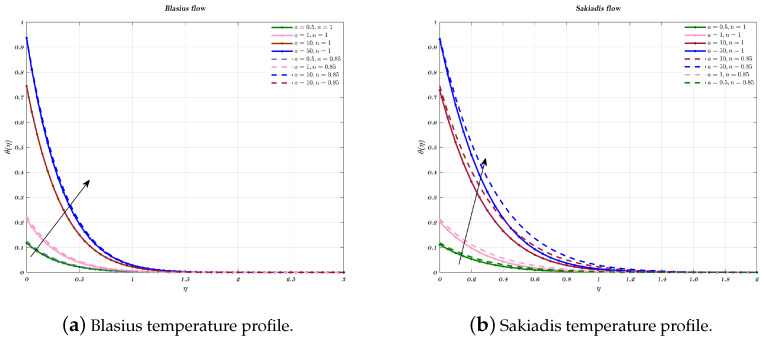
Impact of different values of the convective parameter *a* on temperature profile, for both flows.

Figure 12a,b depict the influence of *a*, which is a convective parameter, on temperature profile in which the effect is seen for both types of nano-fluids. As *a* increases, the surface temperature θ(0) also get rise which shows $h_{f}$ (coefficient of heat transfer) has a relationship that is exactly proportional to the convective parameter. An increase in the convective parameter leads to a decrease in the thermal resistance of the hot surface, which, in turn, causes an increase in the dimensionless surface temperature to be detected. In addition, it is important to notice that as a parameter moves closer and closer to infinity, θ(0) value moves closer and closer to 1. The injection procedure and the technique for an impermeable surface are identical.

### 5.4. Variables Affecting Local Nusselt Number and Skin-Friction Coefficient

**Figure 13:**
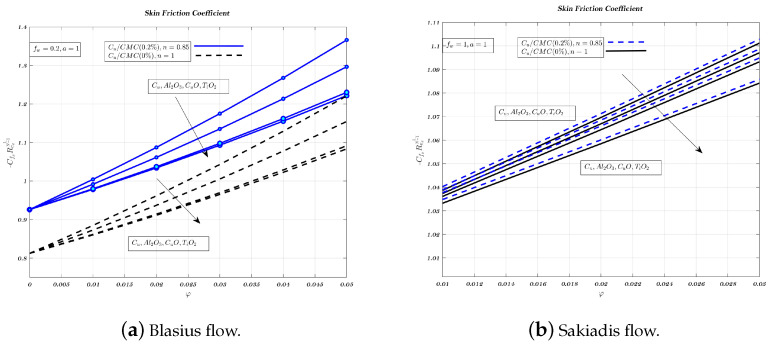
Impact of different nano-particles on skin friction coefficient by considering $f_{w}=0.2$ in case of Blasius flow and $f_{w}=1$ for Sakiadis flow.

**Figure 14:**
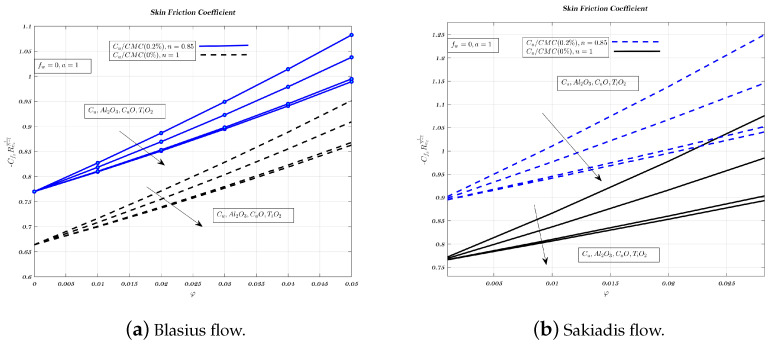
Impact of different nano-particles on skin friction coefficient by considering $f_{w}=0$ in Blasius & Sakiadis flows.

**Figure 15:**
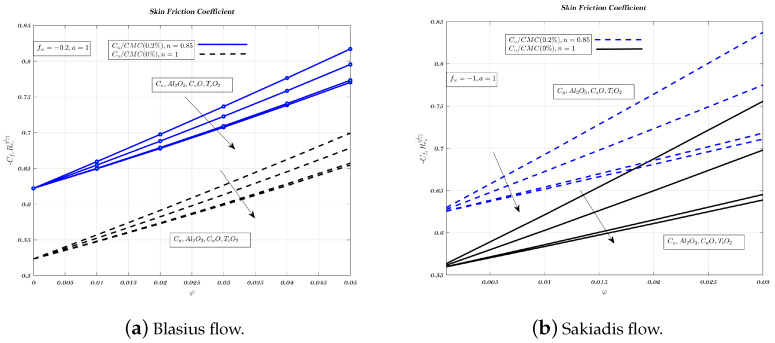
Impact of different nano-particles on skin friction coefficient by considering $f_{w}=-0.2$ in case of Blasius flow and $f_{w}=-1$ for Sakiadis flow.

Different nano-particles have a noticeable effect on the local friction factor for both Newtonian and non-Newtonian nano-fluids, in case of Blasius and Sakiadis flows, as shown in Figure 13a,b. The local friction factor increases when more nano-particles are added to fluids used for transportation. These results highlight the variations in skin friction coefficient over a wide range of VF and magnetic field strengths for both nano-fluids. Therefore, skin friction coefficient founded to increase with both nano-fluids. When the VF of nano-particles is increased, the local friction factor is highest for suction and lowest for injection. Further, the local friction factor of the copper nano-particle is larger than that of other nano-particles. The alumina nano-particle has a lower local friction factor than most other nano-particles. Figure 13, Figure 14 and Figure 15a indicate that in the case of Blasius flow, non-Newtonian nano-fluids outperform Newtonian nano-fluids in terms of heat transfer efficiency [60].

In Sakiadis flow, Figure 13, Figure 14 and Figure 15b show the influence of VF and parameter related to magnetic field for both nano-fluids on skin friction coefficient. In Sakiadis flow, low-thermal conductivity non-Newtonian nano-fluids have a greater heat transfer rate than Newtonian nano-fluids which is true despite the identical amount of particles in both nano-fluids [5,10,61]. Therefore, with respect to $f_{w}$ the influence of nano-particle type on heat transfer rate and skin friction is less significant for the impermeable surface, as shown in Figure 14a,b, while its value is lowest for injection, from Figure 15a,b.

**Figure 16:**
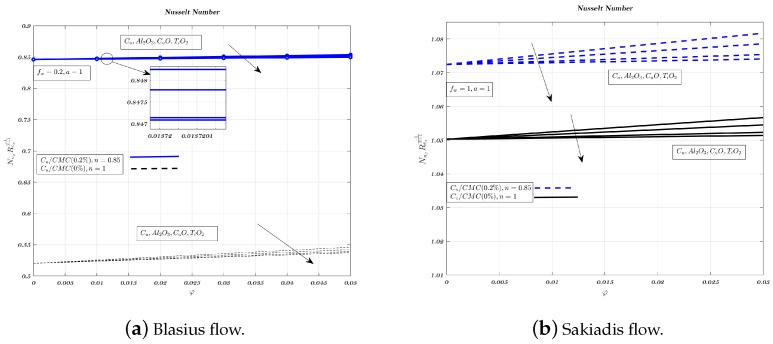
Impact of different nano-particles on Nusselt number by considering $f_{w}=0.2$ in case of Blasius flow and $f_{w}=1$ for Sakiadis flow.

**Figure 17:**
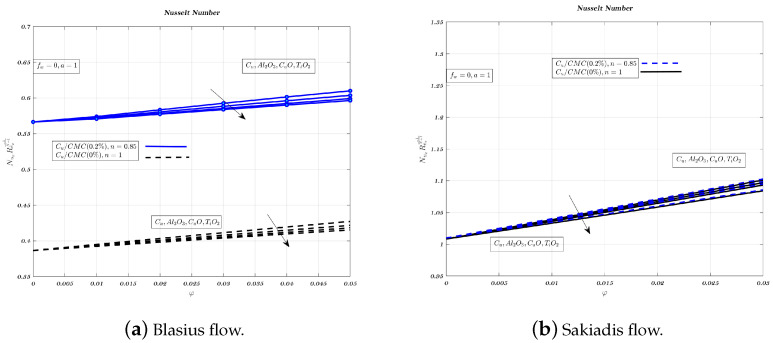
Impact of different nano-particles on Nusselt number by considering $f_{w}=0$ in Blasius & Sakiadis flows.

**Figure 18:**
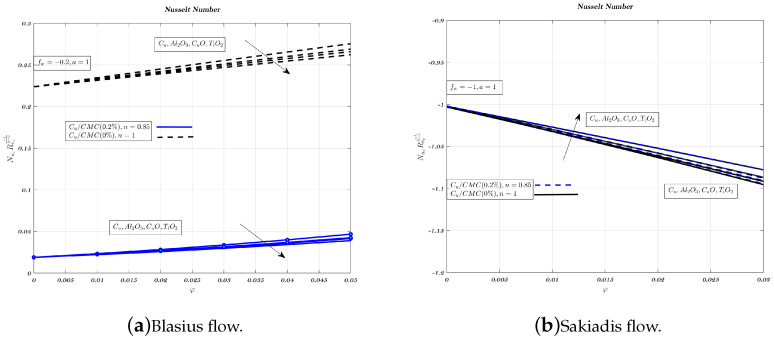
Impact of different nano-particles on Nusselt number by considering $f_{w}=-0.2$ in case of Blasius flow and $f_{w}=-1$ for Sakiadis flow.

Figure 16, Figure 17 and Figure 18a,b show the impact of nano-particles on impermeable surfaces, suction, and injection. Non-Newtonian fluids increase the Nusselt number when suction and an impermeable surface are present. When injected, non-Newtonian nano-fluids reduce heat transfer. Therefore, mixing the carrying fluid with nano-particles of aluminium oxide or titanium dioxide reduces heat transfer and improves suction. Suction is more favourable for heat transfer than the other two situations in terms of Nusselt number and temperature gradient [32]. Aluminium oxide has greater heat conduction than cupric oxide, but lower heat transfer characteristics [62]. In both kinds of nanofluids, increasing volume fraction and magnetic interaction parameter reduces Nusselt number. Low-thermal conductivity alumina nano-particles have a greater heat transfer rate than copper nano-particles in Sakiadis flow.

## 6. Conclusions

This study examined the boundary layer flow of Newtonian and non-Newtonian nano-fluids across a permeable flat plate in a laminar steady state for Blasius and Sakiadis flows. The numerical solution to the governing partial differential equations was achieved by translating them to ordinary differential equations (ODEs) using the similarity approach. These ODEs, then numerically solved. Alumina, copper, titanium, and cupric oxide nano-particles were used in the research in which CMC-water served as the base fluid. Some of the study’s most significant findings are as follows:

- For suction and impermeable surfaces, nano-particles increase fluid velocity in case of Blasius flow, whereas for injection the tendency is reversed for Sakiadis flow. Also, increasing suction reduced boundary layer thickness.
- Large nano-particle VF reduced plate surface temperature for impermeable surfaces by using fluid which is related to non-Newtonian. For suction, nano-particle volume fraction does not affect the temperature which is on the surface in case of low value of *n* while it rise up due to parameter related to suction which affects the surface temperature and thermal boundary layer thickness.
- $Al_{2}O_{3}$ has lower heat transfer than CuO despite better heat conduction.
- Nano-particle volume fraction does not always boost heat transfer. In suction, increasing $Al_{2}O_{3}$ and $TiO_{2}$ nano-particles reduced heat transfer.
- Non-Newtonian fluid injection reduces heat transfer and increases friction.
- In circumstances that are otherwise identical, the value of the local friction factor is found to be greater for non-Newtonian nano-fluids than it is for Newtonian fluids.
- When compared to other nano-particles, the copper nano-particle has a local friction factor that is much greater but in case of alumina nano-particles, it is much lower.
- The impact of the magnetic field in the Blasius flow is to decrease the temperature while increasing the skin friction coefficient, velocity and reduced Nusselt number for both non-Newtonian & Newtonian nano-fluids. On the other hand, this pattern runs in the opposite direction when Sakiadis flow is considered for either the non-Newtonian & Newtonian nano-fluids.
- In Blasius flow, copper nano-particles enhance nano-fluid velocity, whereas alumina nano-particles have the opposite effect. Sakiadis flow over velocity has the opposite effect. In Blasius flow, increasing φ increases the skin friction coefficient, whereas in Sakiadis flow, both non-Newtonian and Newtonian nano-fluids reverse this tendency.
- Non-Newtonian & Newtonian nano-fluids have greater values for both the skin friction coefficient and the reduced Nusselt number in Blasius flow but for Sakiadis flow, such tendency is inverted.

## Figures and Tables

**Table 1:** Thermal conductivity of nano-fluids from previous findings.

Researchers	Nano-Particle Size & Shape	Base-Fluids	Observations
Eastman et al. [3]	Cu Spherical	Water	Vol. Fraction 5 to 20%, improvement 10 to 250%.
Murshed et al. [54]	$TiO_{2}$ Spherical (15 nm)	Water	Vol. Fraction 1 to 5%, improvement 18 to 29.7%.
Chen et al. [52]	$TiO_{2}$ Tube shape	Water	Vol. Fraction 0.25 to 0.6%, improvement 2.5 to 4%.
Das et al. [53]	$Al_{2}O_{3}$ Spherical (38.4 nm)	Water	Vol. Fraction 1 to 4%, improvement 2 to 8%.

**Table 2:** Nano-fluid heat transfer experiment findings.

Researchers	Nano-Particle Size & Shape	Base-Fluids	Observations
Das et al. [53]	$Al_{2}O_{3}$ Spherical (38.4 nm)	Water	Not noticeable rise in Nu
Wen et al. [46]	$Al_{2}O_{3}$ Spherical (30 nm)	Water	Vol. Fraction 0.6 %, Re = 25 to 155, rise in *h* 1.3 to 12.5 %.
Zeinali et al. [1]	$Al_{2}O_{3}$ Spherical (20 nm)	DI Water	Vol. F. 0.2 to 3.0 %, Re = 650 to 2050, rise in *h* 10 to 35 %.
Yang et al. [45]	$Al_{2}O_{3}$ Spherical (20 nm to 56 nm)	Water	Vol. F. 0.2 to 1.0 %, Re = 22 to 900, rise in *h* 3 to 59 %.

**Table 3:** Typical viscosity values for a base fluid (BF) [58].

Physical Characteristics	BF (0%)	BF (0.1%)	BF (0.2%)	BF (0.3%)
***n***	1	0.91	0.85	0.81
** $K/Ns^{n}m^{-2}$ **	0.000855	0.006319	0.017540	0.0313603

**Table 4:** Nano-particles & base fluid thermo-physical characteristics [59].

Physical Characteristics	CMC/Water (0 to 0.3%)	${\mathrm{Al}}_{2}O_{3}$	Ti$O_{2}$	Cu	CuO
**ρ/kg$m^{-3}$**	997.1	8933	3970	6500	4250
**k/W$m^{-1}K^{-1}$**	0.613	400	40	20	8.9538
**$C_{p}$/J$kg^{-1}K^{-1}$**	4179	385	765	535.6	686.2

## Data Availability

Not applicable.
